# Detection of Antiviral Tissue Responses and Increased Cell Stress in the Pancreatic Islets of Newly Diagnosed Type 1 Diabetes Patients: Results From the DiViD Study

**DOI:** 10.3389/fendo.2022.881997

**Published:** 2022-07-26

**Authors:** Lars Krogvold, Pia Leete, Ida M. Mynarek, Mark A. Russell, Ivan C. Gerling, Nataliya I. Lenchik, Clayton Mathews, Sarah J. Richardson, Noel G. Morgan, Knut Dahl-Jørgensen

**Affiliations:** ^1^ Pediatric Department, Oslo University Hospital, Oslo, Norway; ^2^ Faculty of Odontology, University of Oslo, Oslo, Norway; ^3^ Institute of Biomedical and Clinical Sciences, University of Exeter Medical School, Exeter, United Kingdom; ^4^ Faculty of Medicine, University of Oslo, Oslo, Norway; ^5^ Department of Medicine, University of Tennessee Health Science Center, Memphis, TN, United States; ^6^ Department of Pathology, University of Florida, Gainesville, FL, United States

**Keywords:** biopsy, enterovirus, gene expression, MDA5, MxA, pancreas, PKR, Type 1 diabetes

## Abstract

**Aims/hypothesis:**

The Diabetes Virus Detection (DiViD) study has suggested the presence of low-grade enteroviral infection in pancreatic tissue collected from six of six live adult patients newly diagnosed with type 1 diabetes. The present study aimed to compare the gene and protein expression of selected virally induced pathogen recognition receptors and interferon stimulated genes in islets from these newly diagnosed type 1 diabetes (DiViD) subjects vs age-matched non-diabetic (ND) controls.

**Methods:**

RNA was extracted from laser-captured islets and Affymetrix Human Gene 2.0 ST arrays used to obtain gene expression profiles. Lists of differentially expressed genes were subjected to a data-mining pipeline searching for enrichment of canonical pathways, KEGG pathways, Gene Ontologies, transcription factor binding sites and other upstream regulators. In addition, the presence and localisation of specific viral response proteins (PKR, MxA and MDA5) were examined by combined immunofluorescent labelling in sections of pancreatic tissue.

**Results:**

The data analysis and data mining process revealed a significant enrichment of gene ontologies covering viral reproduction and infectious cycles; peptide translation, elongation and initiation, as well as oxidoreductase activity. Enrichment was identified in the KEGG pathways for oxidative phosphorylation; ribosomal and metabolic activity; antigen processing and presentation and in canonical pathways for mitochondrial dysfunction, oxidative phosphorylation and EIF2 signaling. Protein Kinase R (PKR) expression did not differ between newly diagnosed type 1 diabetes and ND islets at the level of total RNA, but a small subset of β-cells displayed markedly increased PKR protein levels. These PKR+ β-cells correspond to those previously shown to contain the viral protein, VP1. RNA encoding MDA5 was increased significantly in newly diagnosed type 1 diabetes islets, and immunostaining of MDA5 protein was seen in α- and certain β-cells in both newly diagnosed type 1 diabetes and ND islets, but the expression was increased in β-cells in type 1 diabetes. In addition, an uncharacterised subset of synaptophysin positive, but islet hormone negative, cells expressed intense MDA5 staining and these were more prevalent in DiViD cases. MxA RNA was upregulated in newly diagnosed type 1 diabetes vs ND islets and MxA protein was detected exclusively in newly diagnosed type 1 diabetes β-cells.

**Conclusion/interpretation:**

The gene expression signatures reveal that pathways associated with cellular stress and increased immunological activity are enhanced in islets from newly diagnosed type 1 diabetes patients compared to controls. The increases in viral response proteins seen in β-cells in newly diagnosed type 1 diabetes provide clear evidence for the activation of IFN signalling pathways. As such, these data strengthen the hypothesis that an enteroviral infection of islet β-cells contributes to the pathogenesis of type 1 diabetes.

## 1 Introduction

The possible influence of viral infections in the development of type 1 diabetes was first postulated by Harris as long ago as 1899, who described a person in whom diabetes developed soon after a bout of mumps (1). Since then, numerous studies have addressed the possible role of viruses as causative agents in type 1 diabetes and, in particular, it has emerged that disease development is strongly associated with infection by enteroviruses. A meta-analysis of such evidence concluded that a clinically significant association exists between enterovirus infection (detected with molecular methods in blood samples) and the occurrence of islet autoantibodies (odds ratio 3.7), as well as between enterovirus infection and the onset of clinical type 1 diabetes (odds ratio 9.8) (2). This conclusion has been reinforced in more recent reviews of the wider literature (3). Enteroviruses (in particular, Coxsackieviruses B4 and B5) have been isolated from the post-mortem pancreas of individuals who had type 1 diabetes on two occasions (4, 5) and their role in disease pathogenesis was reinforced by the demonstration that inoculation of the isolated viruses into mice causes diabetes (4). Additionally, enteroviral-RNA has been detected in blood samples taken from patients with newly diagnosed diabetes (6–8) and prospective studies have suggested that damage to islet cells may be initiated and/or the progression to clinical type 1 diabetes accelerated by enteroviral infection (9, 10).

In the DiViD study, pancreatic specimens of optimal quality have been collected, with written consent, from six live patients (age 24-35 years) with newly diagnosed type 1 diabetes (11). We have previously reported the presence of enteroviral capsid protein 1 (VP-1) in the islets of all six of these patients, and viral RNA was isolated from four of six. These results indicate that a low-grade, possibly chronic, enteroviral infection exists in the islets of such individuals and that this may precede the onset of type 1 diabetes (12). In support of this proposal and, in agreement with previous reports (13, 14), we have shown a marked upregulation of HLA class I at the level of both gene and protein in the insulin-containing islets of DiViD patients, and this may be indicative of a local release of antiviral interferons (15). HLA class I proteins present antigens to cytotoxic T-cells and they are present on all nucleated cells. In type 1 diabetes upregulation of HLA class I is strongly associated with an increased expression of the interferon-responsive transcription factor, signal transducer and activator of transcription 1 (STAT1), a critical protein involved in mediating interferon response to viruses (16). It is, therefore, a reasonable working hypothesis that both the hyperexpression of HLA class I and the upregulation of STAT1 observed in the β-cells of type 1 diabetes donors occur as a consequence of viral infection (15), and that both features serve as “viral footprints” in the endocrine pancreas.

The objectives of the present study were to explore whether additional antiviral responses are also detectable in the islets of Langerhans of individuals with type 1 diabetes. This was achieved by extracting RNA directly from islets of Langerhans using laser capture microdissection and then employing global gene expression profiling as a tool to gain insight into the biological and pathological processes associated with the onset of type 1 diabetes. Secondly, led by the RNA data, the expression of three antiviral tissue response proteins (Interferon-inducible Myxovirus resistance Protein A (MxA), Protein kinase R (PKR) and Melanoma differentiation-associated protein 5 (MDA5) was examined in islets from DiViD subjects and compared with that in islets from similarly aged non-diabetic nPOD-controls (17).

## 2 Materials and Methods

### 2.1 Patient Samples

Pancreatic samples from the six type 1 diabetic patients included in the DiViD study were used in the present investigation. The DiViD study was approved by The Norwegian Governments Regional Ethics Committee. Written informed consent was obtained from all cases after oral and written information from the diabetologist and the surgeon separately. Three women and three men, three to nine weeks after diagnosis, (aged 24-35 years) participated. Detailed clinical characteristics have been described (11).

Six otherwise healthy, non-diabetic organ donors from within the network of Pancreatic Organ Donors (nPOD) biobank were included as controls for immunofluorescent examinations, and the same 6 plus an additional 12 cases were included as controls for the islet laser-capture experiments (**Supplementary Table 1**).

### 2.2 Laser-Capture Microdissection and RNA Extraction

Eight µm thick cryo-sections from the DiViD-cases and the 18 nPOD controls were fixed and dried. 10-20 randomly chosen islets were captured from each section by LCM. The same islets were captured from six consecutive sections. RNA of optimal quality for further analyses was obtained from DiViD-cases 2-6. We have previously described isolation of RNA and obtaining transcriptome data on the Affymetrix Human Gene 2.0 ST arrays (15). Analysis and mining of the Affymetrix array data has been described previously (18).

### 2.3 Immunofluorescent Staining

4μm formalin fixed paraffin embedded sections were obtained from 6/6 DiViD pancreatic biopsies and six similarly aged nPOD controls. The presence and localisation of viral response proteins was then examined by combined immunofluorescent labelling. In brief, sections were dewaxed by submersion in Histoclear™ (Fisher Scientific, UK); antigens were unmasked by heating sections for 20 minutes under pressure in 10mM Citrate (pH 6) and non-specific binding was ameliorated by incubating sections in 5% normal goat serum (Vector, UK). Sections were then incubated with primary antibodies specifically directed against the viral response proteins of interest, in combination with antibodies targeting the various islet hormones (described in **Supplementary Table 2**). Antigens were visualized using fluorescently labelled secondary antibodies (AlexaFluor; Invitrogen, UK) with or without tyramide signal amplification (TSA) (Life Technologies, UK). Islet images were collected and analyzed using a Leica (DM4000) microscope and associated proprietary software (Leica LASX).

## 3 Results

### 3.1 Gene Expression Profiles

After filtering, statistical analysis identified 500 differentially expressed genes in micro dissected islets when comparing the five DiViD-cases employed vs 18 controls (Student’s t-test, p<0.001, fold change >1.1 and false discover rate < 0.1%). Among these 500 genes, several Gene Ontologies (GO) were enriched significantly (**Table 1**). These included ontologies for viral reproduction/infectious cycles, translation elongation/initiation, oxidoreductase activity and mitochondrial and ribosomal genes. Interrogation of the gene list using the KEGG database (http://www.genome.jp/kegg/pathway.html), as well as the Ingenuity Pathway System (https://www.qiagenbioinformatics.com/products/ingenuity-pathway-analysis/), yielded evidence for significant enhancement of pathways relating to mitochondrial function, oxidative phosphorylation, ribosomes, metabolism, antigen processing and presentation and EIF2 signaling among the most significantly enriched pathways (**Table 2**). Analysis of the promoter regions of the 500 genes demonstrated enrichment of genes regulated by transcription factors involved in cell growth, mitochondrial function/regulation, and inflammation and interferon responses. A complete list consisting of all 500 genes differentially expressed is showed in **Supplementary Table 3**. The table includes transcripts cluster ID, corrected p-value, fold change, entrez gene and gene symbol.

**Table 1:** Gene Ontology analyses of gene expression in DiViD cases compared to controls.

Ontologies that showed significant enrichment in cases compared to non-diabetic controls		
Biological processes:		
Viral reproduction genes:	37 genes	adjP=5.85e-09
Viral reproductive process	33 genes	adjP=2.50e-09
Viral infectious cycle	24 genes	adjP=1.80e-11
Viral genome expression	21 genes	adjP=9.27e-12
Viral transcription	21 genes	adjP=9.27e-12
Metabolic processes:		
Translation genes	43 genes	adjP=7.70e-10
Translational initiation	23 genes	adjP=1.94e-13
Translational termination	15 genes	adjP=1.69e-09
Translational elongation	18 genes	adjP=1.80e-11
Nuclear-transcribed mRNA catabolic process		
Nonsense-mediated decay	16 genes	adjP=5.85e-09
Molecular function:		
RNA binding	40 genes	adjP=7.18e-09
Ribosome binding	5 genes	adjP=1.70e-03
Translation factor activity, nucleic acid binding	8 genes	adjP=1.20e-03
Structural molecule activity	33 genes	adjP=1.15e-08
Structural constituent of ribosome	26 genes	adjP=8.35e-18
NADH dehydrogenase activity	5 genes	adjP=1.08e-02
ATPase activity	14 genes	adjP=1.61e-02
Cellular component:		
Macromolecular complex	107 genes	adjP=2.66e-11
Intracellular	230 genes	adjP=1.11e-11
Intracellular organelle part	154 genes	adjP=2.99e-11
Cytoplasm	194 genes	adjP=1.95e-13
Cytoplasm part	157 genes	adjP=7.07e-12
Ribonucleoprotein complex	38 genes	adjP=1.37e-13
Ribosomes	29 genes	adjP=4.36e-18
Ribosomal subunit	22 genes	adjP=4.42e-15
Cytosolic ribosome	16 genes	adjP=9.06e-12

**Table 2:** Pathway and Transcription factor analysis of gene expression in DiViD cases compared to controls.

Enrichment in the following KEGG pathways			
Oxidative phosphorylation (27/132 genes)	p-value 1.08 E-27		
Ribosome (20/92 genes)	p-value 2.08 E-21		
Metabolic Pathways (53/1130 genes)	p-value 6.71 E-21		
Enrichment of these canonical pathways:	
Mitochondrial dysfunction (30/171 genes)		p-value 5.74 E-22	
Oxidative phosphorylation (25/109 genes)		p-value 1.67 E-21	
EIF2 Signaling (26/185 genes)		p-value 1.04 E-16	
Antigen presentation (11/37)		p-value 1.62 E-11	
Enrichment of promoter and *de novo* pathways:			
Transcription factors involved in cellular growth/apoptosis:			
Elk-1, 83 genes			p-value 2.40 E-8
Tel-2, 44 genes			p-value 1.93 E-4
Mitochondrial function (GABP), 93 genes			p-value 1.93 E-4
Interferon regulatory factor 7 (IRF7), 44 genes			p-value 9.85 E-4

### 3.2 Antiviral Tissue Responses

#### 3.2.1 MxA

**Figure 1:**
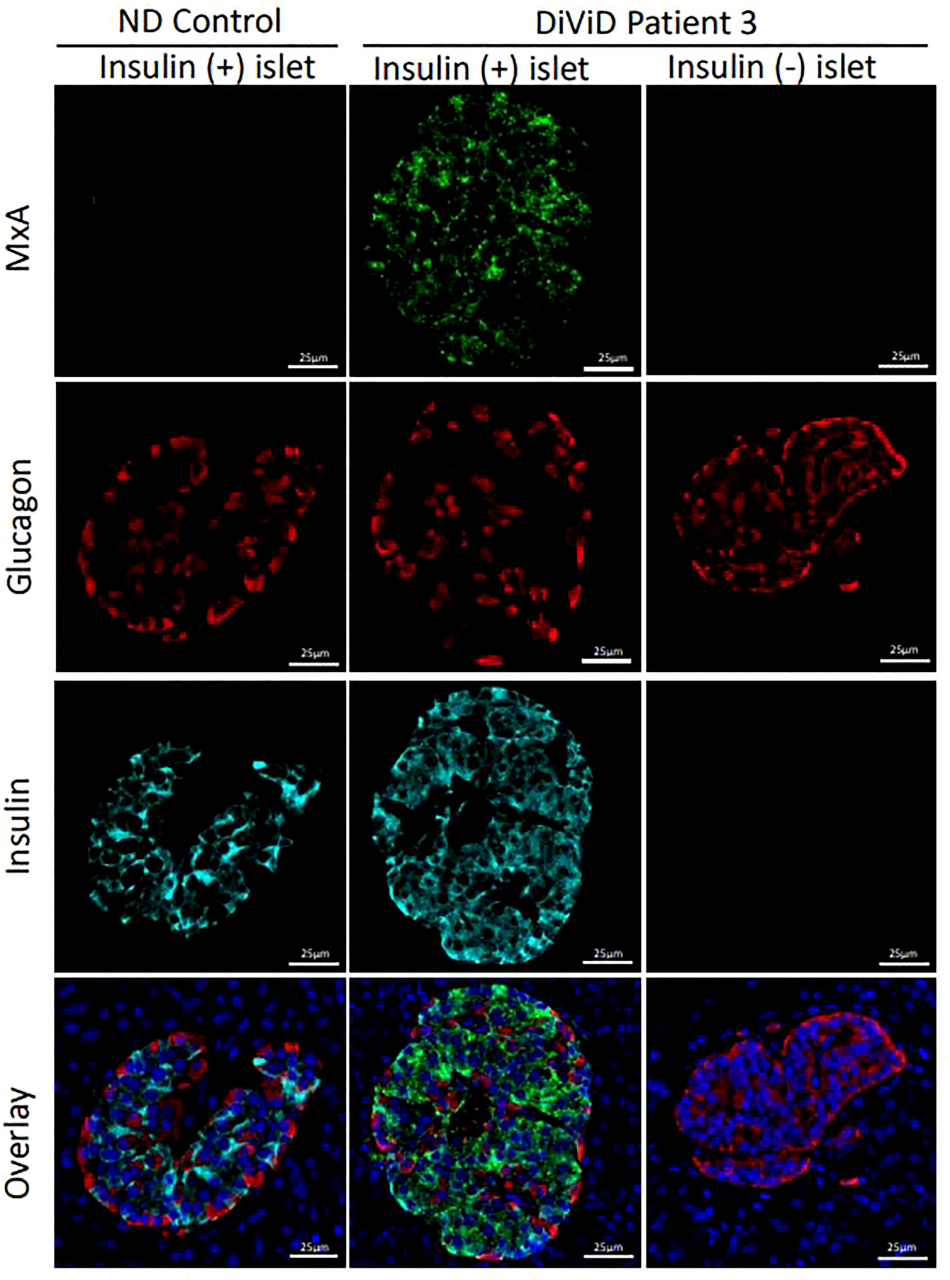
Micrographs illustrating the immunofluorescent staining of representative islets from an individual without diabetes (ND; column 1) and two islets from a DiViD patient with diabetes, either with or without residual beta cells (columns 2 and 3 respectively). Green, MxA; Red, Glucagon; Cyan, Insulin; Blue, DAPI. Scale bars, 25um.

RNA encoding MxA was present at significantly higher levels in the islets of individuals with type 1 diabetes vs ND islets (p < 0.00005). To verify that this also resulted in an increase in MxA protein, immunostaining profiles were examined in 124 type 1 diabetes islets (70 with residual beta cells) vs 26 islets from 6 non-diabetic subjects. MxA was expressed exclusively in type 1 diabetes islets and was detected in 91.4% (64 of 70) of those islets with residual insulin immunopositivity. MxA immunopositivity was restricted only to β-cells and it was not present in other islet endocrine cells (**Figure 1**) and was not found in any insulin-deficient islets in the DiViD subjects nor in the islets of control subjects.

#### 3.2.2 PKR

**Figure 2:**
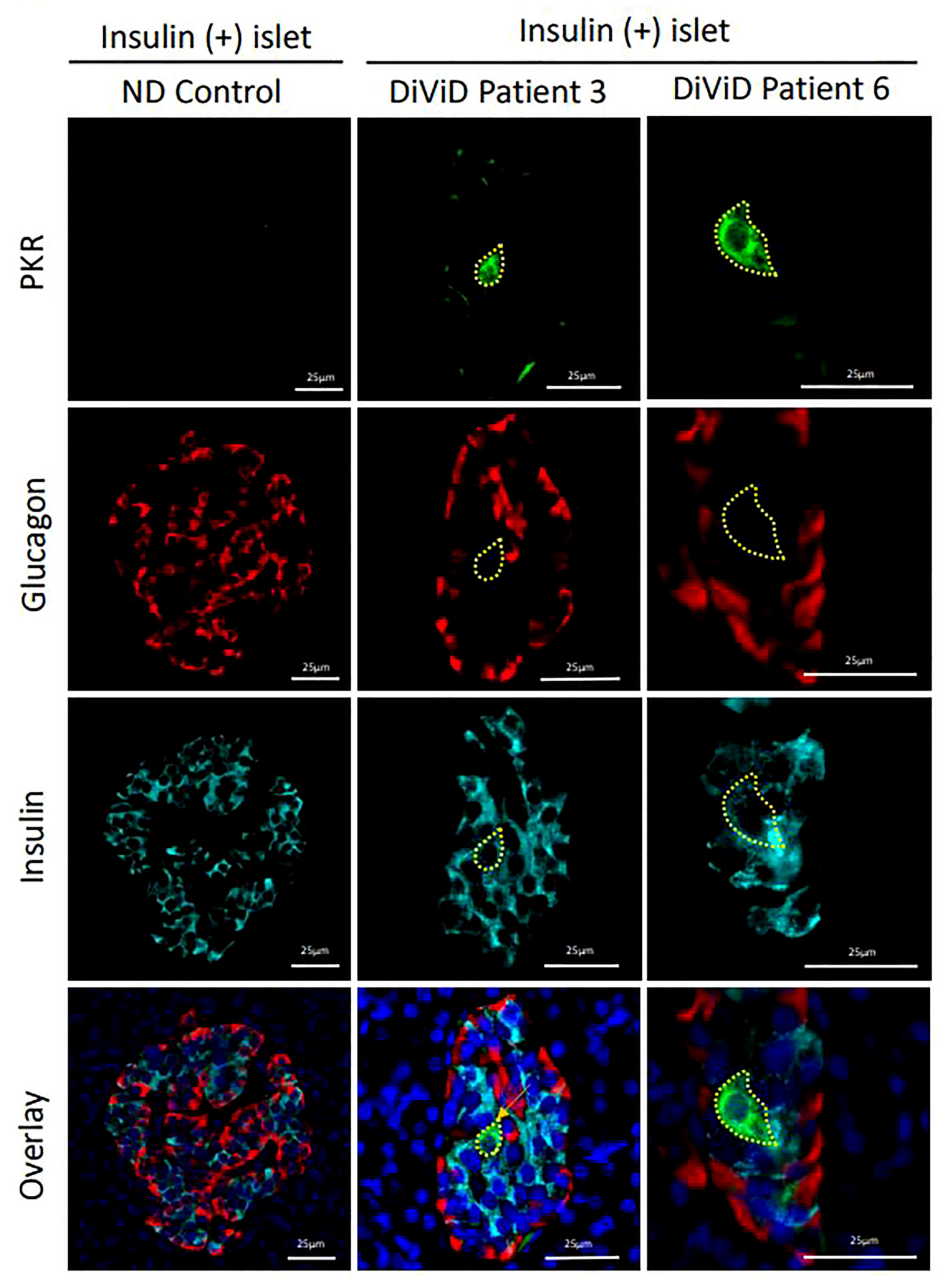
Micrographs showing immunofluorescence images of representative islets from an individual without diabetes (ND; column 1) and from two different DiViD patients with diabetes (columns 2 & 3). PKR positive beta cells are indicated (yellow lines). Green, PKR; Red, Glucagon; Cyan, Insulin; Blue, DAPI. Scale bars, 25um.

No differences in RNA expression of *EIF2AK2* (which encodes protein kinase R) was observed between whole islets from the DiViD subjects and controls, (p=0.708). However, when monitoring protein expression, it was evident that a small subset of β-cells in each of the DiViD subjects displayed markedly increased PKR levels (**Figure 2**). Such cells were only seen in residual insulin-containing islets and are consistent with earlier work demonstrating that PKR is present at high levels in beta cells that also contain the enteroviral capsid protein, VP1 (19).

#### 3.2.3 MDA5

RNA encoding MDA5 was significantly increased in type 1 diabetes islets compared to controls (p < 0,005). However, marked differences in expression were noted between subjects, with the highest levels found in one individual (DiViD case 5) while MDA5 mRNA was elevated less strongly in the remainder.

**Figure 3:**
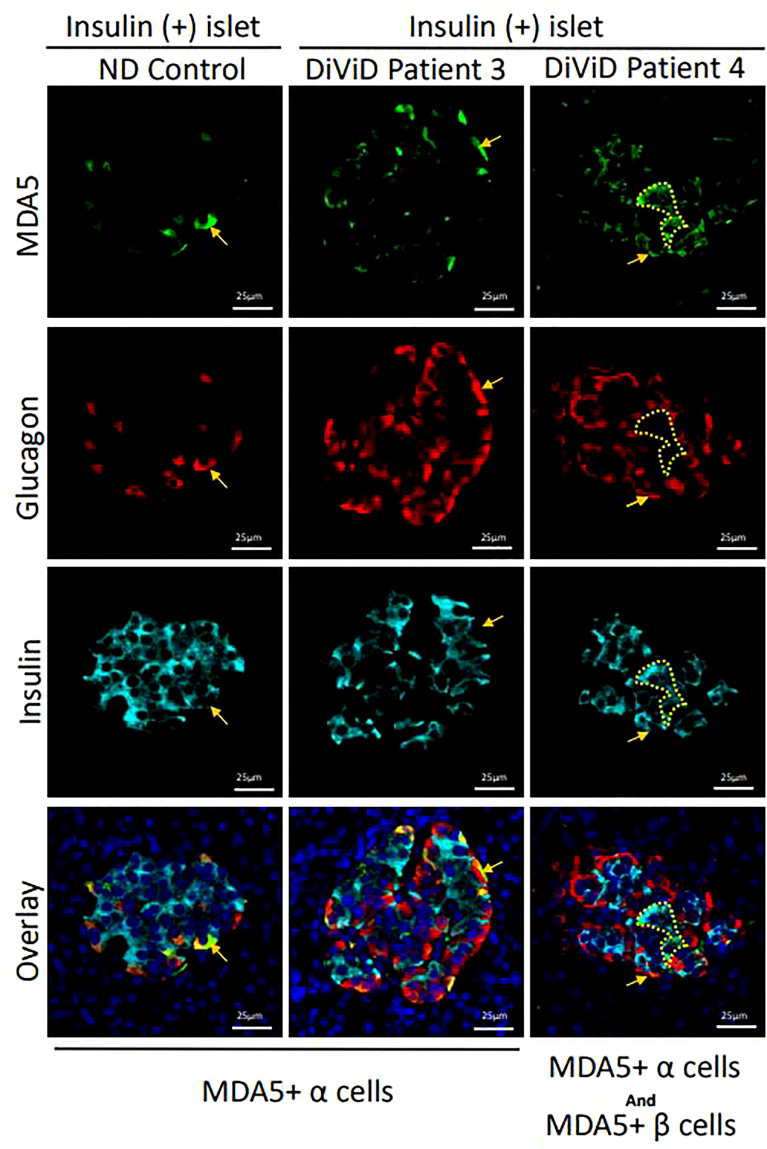
Micrographs showing immunofluorescent images of a representative islet from an individual without diabetes (ND; column 1) and from two DiViD patients with diabetes (columns’ 2-3). MDA5+ alpha cells are indicated with arrows and MDA5+ beta cells with dotted lines (columns 2 & 3). Green, MDA5; Red, glucagon; Cyan, Insulin; Blue, DAPI. Scale bars, 25um.

**Figure 4:**
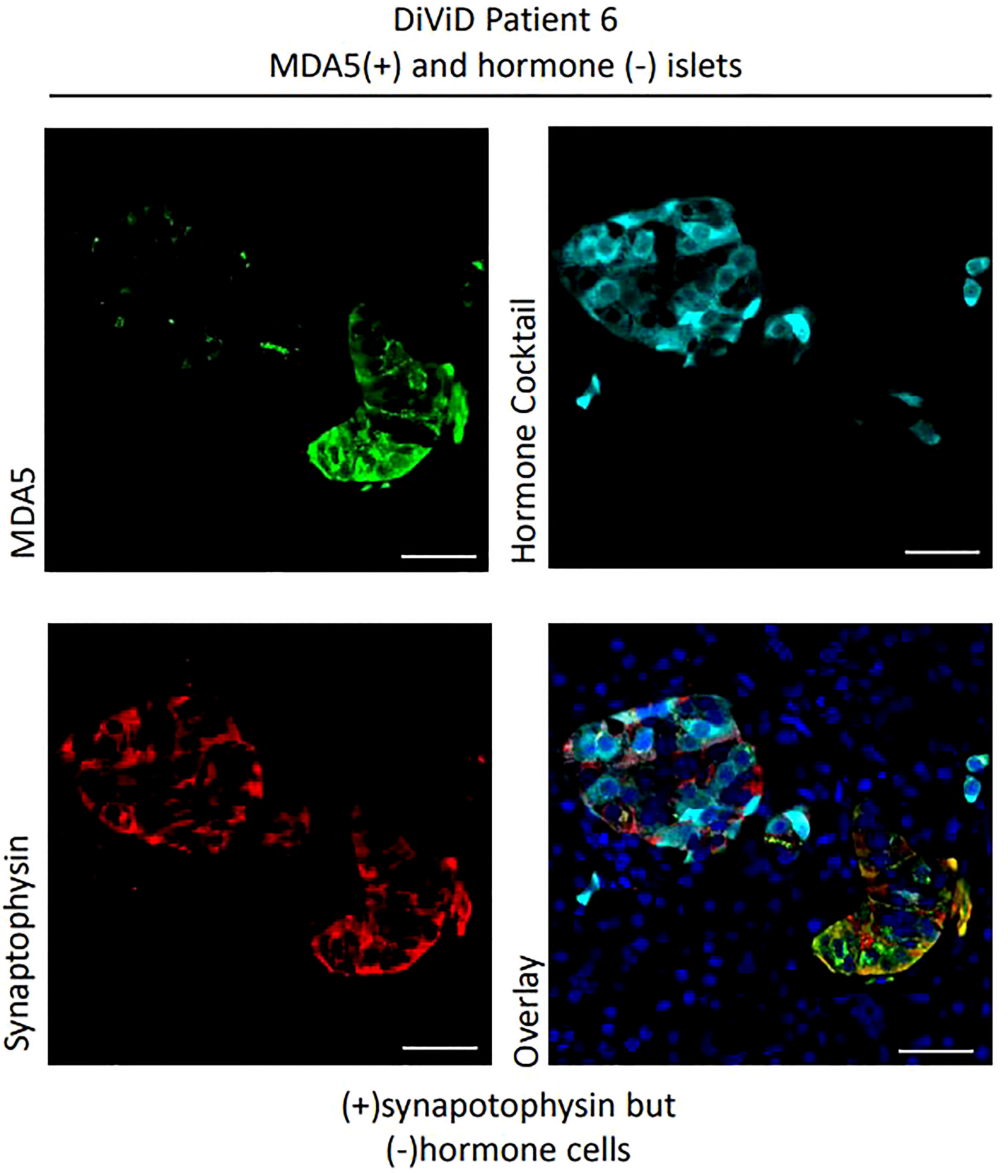
Examples of MDA5+ cells which are synaptophysin + but hormone negative. Green, MDA5; Red, synaptophysin; Cyan, hormone cocktail; insulin, glucagon and somatostatin. Scale bars, 25um.

Among 124 islets examined histologically, MDA5 protein was highly expressed in some α-cells of all DiViD subjects and controls. The protein was rarely detected in the β-cells of controls (it was detected weakly in only 1 of 25 islets imaged), however among the 33 ICIs imaged in the DiViD subjects, 25 (75.8%) exhibited a marked upregulation of MDA5 within β-cells (**Figures 3**, **4**), and this phenomenon was seen across all individuals. The response was however not seen in all beta cells but was restricted to a subpopulation within any given ICI (**Figures 3**, **4**). In addition, a further subset of islet endocrine (synaptophysin+) cells were seen to display elevated and punctate expression of MDA5 but these were not immunopositive for either somatostatin, insulin or glucagon. Although not common, these cells and clusters were seen in six of six DiViD cases (**Figure 4**).

## 4 Discussion

We have used both RNA expression analysis and protein immunodetection to monitor the induction of antiviral signaling in the islets of DiViD vs control subjects of equivalent ages. The data mining process revealed significant enrichment of several gene ontologies linked to viral infection in the DiViD tissue; findings which accord with gene and protein expression data revealing elevated levels of MxA and MDA5, as well as induction of PKR expression in a subpopulation of beta cells in the DiViD subjects. As such, the results reported here strengthen and expand our previous observations implying the presence of a sustained low-grade enteroviral infection in the pancreatic islet tissue of DiViD cases (12). These findings also correlate with the previously noted upregulation of STAT1 and HLA class 1 described in the islets of these patients (15). Furthermore the results are in line with the recently published results from the nPOD study reporting from organ donors cases with prediabetes and variable duration of type 1 diabetes (20).

When considering the RNA expression data presented here, it is important to emphasize that, islets harvested by laser microdissection from the DiViD-cases were not pre-selected according to their insulin content or the extent of inflammation; rather, all available islets within any given section were captured to deliver an unbiased analysis. Inevitably, therefore, the islets comprised a heterogeneous population having variable proportions of alpha and beta cells, as well as differential immune cell infiltration (21). This will have reduced the contribution made by any alterations in gene expression that occurred selectively in beta cells or in specific subpopulations of islets, thereby implying that the changes reported here are likely to represent an underestimate of the true magnitude of the responses. In addition, due to technical difficulties, RNA of appropriate quality could not be extracted from DiViD case 1.

It should also be emphasized that the data-mining technique is unbiased and observational, such that it is not bound by prior hypotheses. While this is a clear strength, it must also be acknowledged that it carries the risk that false positive differences might be highlighted. Conscious of this limitation, we chose a stringent level of significance for inclusion of gene expression changes (p<0.001). The corollary to these considerations is that we may have thereby omitted certain smaller scale differences in gene expression occurring between the DiViD subjects and controls and, in support of this, we noted that reducing the stringency (top>0.05) results in an increase in the number of differentially expressed genes from 500 to > 21 000. Thus, the application of stringent statistical limits gives confidence that any differences described here, represent genuine differences between the two groups.

The observed enhancement of genes involved in mitochondrial and ER stress pathways is consistent with previously published data. Islet cells from individuals with type 1 diabetes display a partial ER stress response, with evidence of the induction of some, but not all, components of the unfolded protein response (22). This could occur because of enteroviral infection (although other environmental factors such as reactive oxygen species might be involved) but the enrichment of several biological gene ontologies involving viral reproduction, viral genome expression and viral transcription (**Table 2**) supports the possibility of viral involvement. Strong support for this hypothesis is also provided by the direct demonstration of increased antiviral protein expression in the islets of DiViD cases. MxA is an antiviral response protein, driven by the upregulation of type 1 interferons and STAT1 (23). Hence, MxA serves as a very effective surrogate for the expression of type 1 interferons and it was not observed in any of the islets of individuals without diabetes but was strongly expressed in those with type 1 diabetes. Similarly, PKR is activated and upregulated in response to double-stranded RNA (dsRNA) of viral origin (24) and was found previously in a small subpopulation of VP1 positive cells in the islets of people with type 1 diabetes (19). Thus, an increase in PKR expression also suggests viral infection in islets. Finally, MDA5, encoded by the gene *IFIH1*, is a pattern recognition receptor which senses dsRNA generated during viral replication. In the present work, all three of these proteins were upregulated selectively in the islets of DiViD cases compared to non-diabetic controls. For two of the viral sensor proteins (PKR and MDA5) the increases were restricted only to certain beta cells whereas, for MxA, a much larger proportion of beta cells were responsive. This must imply that different mechanisms are involved in driving the increases (i.e. interferon release is unlikely to be the sole mechanism) but the fact that all three sensors were elevated is consistent with the hypothesis that a sustained enteroviral infection occurs in at least some beta cells in type 1 diabetes. Furthermore, our finding that these collective responses were primarily restricted to beta cells, and were not seen in alpha cells or in the pancreatic exocrine tissue, supports the concept that beta cells are uniquely susceptible to enteroviral infection.

One unexpected outcome of this study was that we also noted the presence of MDA5 positive, but somatostatin/insulin/glucagon immunonegative cells in type 1 diabetes cases. The identity of these cells has not been fully verified but, despite their lack of hormone staining, they were positive for the endocrine cell marker synaptophysin. Thus, it is possible that they could represent a population of dedifferentiating or nascent beta cells that either have lost, or not yet acquired, insulin immunopositivity. This observation in samples from the DiViD cases, using a different antibody against MDA5 (ab4544, Abcam), has recently been published (25). These two findings identifies a potentially important concept, which will require further verification in subsequent studies.

In conclusion, an analysis of islet gene expression signatures in patients with newly diagnosed type 1 diabetes, showing the enrichment of gene ontologies for viral reproduction and the infectious cycle, supports the proposal that enteroviruses are involved in the pathogenesis of type 1 diabetes. The elevated levels of PKR, MxA and MDA5 in type 1 diabetes beta cells, together with our previous studies using these samples which revealed increased HLA-1 and STAT1 in the beta cells, where both nuclear and cytoplasmic was observed, provide firm support for the activation of IFN signaling and other antiviral pathways. As such, these new results strengthen the hypothesis that chronic enteroviral infection contributes to the pathogenesis of type 1 diabetes.

## Data Availability Statement

The datasets presented in this article are not readily available because of the sensitive nature of the data and possible high risks associated with patient confidentiality. Requests to access the datasets should be directed to the authors.

## Ethics Statement

The studies involving human participants were reviewed and approved by The Norwegian Governments Regional Ethics Committee, post@helseforskning.etikkom.no. The patients/participants provided their written informed consent to participate in this study.

## Author Contributions

LK was responsible for clinical coordination and recruitment of patient, data collection, analysis and interpretation, and drafted the manuscript. IM, PL, MR, SR and NM performed, analyzed and interpreted the immunofluorescent analyses. IG, CM and NL were responsible for the laser capture, RNA extraction and subsequent gene expression profile data collection. All authors contributed in analysis and interpretation of the results, and in writing of the manuscript. KD-J was the principal investigator of the study, had the initial idea of the DiViD study, and participated in study design, funding, regulatory issues, international collaboration, data collection, analysis and interpretation, and in writing of the manuscript. All authors edited the manuscript. LK and KD-J are the guarantors of this work and, as such, had full access to all the data in the study and take responsibility for the integrity of the data and the accuracy of the data analysis. All authors contributed to the article and approved the submitted version.

## Funding

The project was funded by South-Eastern Norway Regional Health Authority (Grant to KDJ), The Novo Nordisk Foundation (Grant to KD-J) and through the PEVNET Study Group funded by the European Union’s Seventh Framework Programme [FP7/2007-2013] under grant agreement n°261441 PEVNET. The participants of the PEVNET consortium are described at http://www.uta.fi/med/pevnet/publications.html. Additional support was from a JDRF Career Development Award (5-CDA-2014-221-A-N) to SR, a JDRF research grant awarded to the network of Pancreatic Organ Donors – Virus (nPOD-V) consortium (JDRF 25-2012-516); and an MRC Project Grant MR/P010695/1 awarded to SR & NM. The work was also supported by a grant from National Institute of Health (DK104155), JDRF (JDRF 25-2010-723 & JDRF 47-2013-520) to IG and CM and by the Leona M & Harry B Helmsley Charitable Trust (Grant#2018PG-type 1 diabetes053). This research was performed with the support of the Network for Pancreatic Organ donors with Diabetes (nPOD; RRID : SCR_014641), a collaborative type 1 diabetes research project sponsored by JDRF (nPOD: 5-SRA-2018-557-Q-R) and The Leona M. & Harry B. Helmsley Charitable Trust (Grant#2018PG-type 1 diabetes053). Organ Procurement Organisations (OPO) partnering with nPOD to provide research resources are listed at http://www.jdrfnpod.org//for-partners/npod-partners/

## Conflict of Interest

The authors declare that the research was conducted in the absence of any commercial or financial relationships that could be construed as a potential conflict of interest.

## Publisher’s Note

All claims expressed in this article are solely those of the authors and do not necessarily represent those of their affiliated organizations, or those of the publisher, the editors and the reviewers. Any product that may be evaluated in this article, or claim that may be made by its manufacturer, is not guaranteed or endorsed by the publisher.

## Abbreviations

MDA5, Melanoma differentiation-associated protein 5; MxA, Myxovirus resistance Protein A; ND, non-diabetic; PKR, Protein kinase R; STAT1, signal transducer and activator of transcription 1; VP1, enteroviral capsid protein 1.
