# Supplementary material for: Detection of Antiviral Tissue Responses and Increased Cell Stress in the Pancreatic Islets of Newly Diagnosed Type 1 Diabetes Patients: Results From the DiViD Study
Source: Front Endocrinol (Lausanne). 2022 Jul 26;13:881997. doi: 10.3389/fendo.2022.881997 (PMC9360491; doi:10.3389/fendo.2022.881997)
Supplement: Supplementary file 1 [file Table_1.pdf]

Supplementary table 1

nPOD ID	Sex	Age at death (y)	Immunfluorescent	Laser capture
6010	Female	47		x
6012	Female	68		x
6013	Male	65		x
6019	Male	42		x
6024	Male	21		x
6048	Male	30	x	x
6075	Male	16		x
6099	Male	14		x
6102	Female	45		x
6129	Female	43		x
6140	Male	38	x	x
6162	Male	23	x	x
6165	Female	46		x
6168	Male	51		x
6179	Female	22	x	x
6227	Female	17		x
6229	Female	31	x	x
6251	Female	33	x	x
