# Supplementary material for: Detection of Antiviral Tissue Responses and Increased Cell Stress in the Pancreatic Islets of Newly Diagnosed Type 1 Diabetes Patients: Results From the DiViD Study
Source: Front Endocrinol (Lausanne). 2022 Jul 26;13:881997. doi: 10.3389/fendo.2022.881997 (PMC9360491; doi:10.3389/fendo.2022.881997)
Supplement: Supplementary file 2 [file Table_2.pdf]

Supplementary table 2

Antibody	Vendor	Cat #	Antibody conditions	HIER	Block
MDA5	Abcam	ab69983	1/500 (1h RT TSA)	Citrate pH6	5%NGS
PKR	Abcam	ab32052	1/700 (overnight 4C)	Citrate pH6	5%NGS
MxA	From O Haller	Kindly donated	1/100 (1h RT TSA)	Citrate pH6	5%NGS
Insulin	Dako	A0564	1/343 (1h RT)	Citrate pH6	5%NGS
Glucagon	Abcam	ab10988	1/2000 (1h RT)	Citrate pH6	5%NGS
Somatostatin	Abcam	ab30788	1/200 (overnight 4C)	Citrate pH6	5%NGS
VP1	Dako	M7064	1/1400 (1h RT)	Citrate pH6	5%NGS
