# Supplementary material for: Detection of Antiviral Tissue Responses and Increased Cell Stress in the Pancreatic Islets of Newly Diagnosed Type 1 Diabetes Patients: Results From the DiViD Study
Source: Front Endocrinol (Lausanne). 2022 Jul 26;13:881997. doi: 10.3389/fendo.2022.881997 (PMC9360491; doi:10.3389/fendo.2022.881997)
Supplement: Supplementary file 3 [file Table_3.pdf]

Supplementary table 3

Transcripts Cluster Id	Corrected p-value	Fold change	Entrez gene	Gene symbol
16817790	9,61E-06	1,6053501	226	ALDOA
16906534	9,61E-06	-4,0889874	6772	STAT1
16932300	9,61E-06	1,7018756	7353	UFD1L
16821614	1,06E-05	1,7480265	1327	COX4I1
16965773	1,06E-05	2,0788307	100422937	MIR4275
16977045	1,06E-05	-19,04026	4283	CXCL9
16721715	1,36E-05	1,504834	6157///619562	RPL27A|SNORA3
16664272	2,24E-05	1,8440099	440567///7388	UQCRHL
16771216	2,24E-05	1,9564127	6175	RPLP0
16748751	3,24E-05	1,58976	11171///100653179///100653013	STRAP|LOC100653179|LOC100653013
17100173	3,47E-05	1,8443079	8721	EDF1
16948738	3,97E-05	1,308604	1173	AP2M1
16962264	5,07E-05	1,8260458	64110	MAGEF1
16670673	5,88E-05	1,9787961		
16680274	5,88E-05	1,5172502	54998	AURKAIP1
16693642	5,88E-05	1,8941478	10899	JTB
16701287	5,88E-05	1,532219	159	ADSS
16752223	5,88E-05	2,0632873	29095	ORMDL2
16755487	5,88E-05	-2,782032	100302289	MIR1251
16758549	5,88E-05	1,7651839	51329	ARL6IP4
16833742	5,88E-05	1,8507832	6143	RPL19
16858860	5,88E-05	2,0103533	28974	C19orf53
16869660	5,88E-05	1,8229016	4713	NDUFB7
16935601	5,88E-05	1,4820088		
16973322	5,88E-05	1,6577387	521	ATP5I

17011683	5,88E-05	1,9356555	112495	GTF3C6
17024418	5,88E-05	1,8798299	83443	SF3B5
17092737	5,88E-05	1,4081752	6194	RPS6
17117588	5,88E-05	1,6645302	727803	LOC727803
16800450	6,15E-05	-1,9730647	567	B2M
16662744	6,32E-05	2,1710012	4725	NDUFS5
16969344	6,32E-05	1,9090427	493856	CISD2
16935264	6,47E-05	1,5658576	91582	RPS19BP1
16766260	6,75E-05	1,5567882	506	ATP5B
16678303	6,81E-05	1,632163	375///100500810	ARF1|MIR3620
16765134	6,81E-05	1,6993176	3856	KRT8
16804778	6,81E-05	1,5202425	51335	NGRN
16843511	6,81E-05	-3,4628165	6352	CCL5
17002871	6,81E-05	1,6904979		
17013002	6,81E-05	1,7957641	23593	HEBP2
17060237	6,81E-05	1,6330276	9551///100526740///26024	ATP5J2|ATP5J2-PTCD1
17100697	6,81E-05	-1,859603	4541	ND6
16744684	6,86E-05	1,492851	8882	ZNF259
16726439	6,89E-05	1,7886096	25824	PRDX5
16835589	7,20E-05	2,1396792		
16857353	7,20E-05	1,7645236	25873	RPL36
17056830	7,20E-05	-2,0149221		TRGV10
17059776	7,20E-05	-2,4087741	219285	SAMD9L
16664100	7,30E-05	1,9178879	10327	AKR1A1
16959187	7,30E-05	1,5974346	11222	MRPL3
17027330	7,30E-05	-1,551038	3135	HLA-G

17037658	7,44E-05	-1,5491024	3135	HLA-G
16856153	7,51E-05	2,461526	10907	TXNL4A
16808363	7,62E-05	1,8066527	100302254	MIR1282
16872267	7,66E-05	1,9915428	2091	FBL
16680348	8,19E-05	1,650894	29101	SSU72
16734840	8,19E-05	-1,8833305	3043	HBB
16782122	8,19E-05	-6,4633994	28738///28755///28663	TRAJ17|TRAC|TRAV20
16840284	8,19E-05	1,8364732	708	C1QBP
16856476	8,19E-05	2,1889434	513	ATP5D
16857429	8,19E-05	1,6084784	8192	CLPP
16972305	8,19E-05	1,5875939	1182	CLCN3
16830182	8,52E-05	1,9689989	84817	TXNDC17
16959760	8,52E-05	1,5988265	9276	COPB2
16825923	8,77E-05	1,4193965	79759	ZNF668
16814488	8,82E-05	1,9632945	10273	STUB1
16977912	8,82E-05	2,2758563	84992	PIGY
17078480	8,82E-05	2,1424837	28957///7163	MRPS28|TPD52
16690720	8,98E-05	1,4544479	10542	HBXIP
16748527	8,98E-05	2,186047	387841	RPL13AP20
17000800	8,98E-05	1,7978046	4695	NDUFA2
17001654	8,98E-05	-2,6456656	972	CD74
16855065	9,02E-05	1,8298297	51124	IER3IP1
17098322	9,02E-05	1,3364692	11224	RPL35
16700888	9,08E-05	2,2007434	4811	NID1
17084403	9,08E-05	2,0360258	54926	UBE2R2
16844119	9,55E-05	1,4891949		

16871051	9,88E-05	1,7001603	7386	UQCRFS1
16872640	1,00E-04	2,3451931	10567	RABAC1
16873329	1,00E-04	-3,1335533	2354	FOSB
17036605	1,00E-04	1,4326719		
16871359	1,04E-04	1,6027057	93099	DMKN
16885526	1,07E-04	1,6840968	92856	IMP4
16910309	1,07E-04	-1,3880244		
16693515	1,09E-04	1,6542281	3608	ILF2
16942906	1,09E-04	1,6053895	25978	CHMP2B
17031178	1,09E-04	-2,2270894	3118///3117///100509457///100507718	HLA-DQA2|HLA-DQA1|LOC100509457|LOC100507718
17093090	1,09E-04	-2,0662997	23586	DDX58
16913198	1,10E-04	1,9078462	140823	ROMO1
17023484	1,18E-04	1,7062277	93663	ARHGAP18
16845259	1,21E-04	1,9855126	28958	CCDC56
17008088	1,25E-04	1,8310621	1337	COX6A1|COX6A1P2
16989130	1,25E-04	1,6114024	27089	UQCRQ
16860047	1,30E-04	1,7289727	51079///374887	NDUFA13|YJEFN3
16873007	1,30E-04	1,9493725	23474	ETHE1
16933630	1,30E-04	1,3315512	8563	THOC5
17049060	1,30E-04	-1,7504992		
17057983	1,30E-04	1,6530797	51142	CHCHD2
16865175	1,33E-04	1,3855374	4696	NDUFA3
16834441	1,48E-04	1,8440464	84313	VPS25
16772733	1,49E-04	1,439239	2802	GOLGA3
16821021	1,49E-04	1,8977748	5713	PSMD7

16825753	1,49E-04	1,5558952	10421	CD2BP2
16866946	1,49E-04	2,1292374	26517	TIMM13
17027390	1,49E-04	1,2975389	135644	TRIM40
17098626	1,49E-04	1,5093346	203	AK1
17104540	1,49E-04	1,4828246	1741	DLG3
16814872	1,49E-04	1,4425532	10607	TBL3
16863946	1,49E-04	1,7298723	10856	RUVBL2
16957738	1,49E-04	1,3622946	8702	B4GALT4
17041260	1,55E-04	-1,9779669	3118///100509457///100507718///3117	HLA-DQA2|LOC100509457|LOC100507718|HLA-DQA1
16666509	1,58E-04	-2,5458012	10561	IFI44
16842237	1,58E-04	7,195089	780853	SNORD3C
16980768	1,61E-04	-1,7801794		
17109706	1,64E-04	1,833016	1964	EIF1AX
17028345	1,65E-04	-2,0689793	3118///3117	HLA-DQA2|HLA-DQA1
17029712	1,66E-04	-2,2845814	3123///3125///3126///100507714///100507709	HLA-DRB1|HLA-DRB3|HLA-DRB4|LOC100507714|LOC100507709
16739117	1,69E-04	1,3864251	746	C11orf10
16765744	1,69E-04	1,3029301	967	CD63
17057174	1,72E-04	1,7589284	5425	POLD2
16869387	1,76E-04	1,8941089	90480	GADD45GIP1
16758182	1,77E-04	-2,2803228		
17039914	1,77E-04	-2,2652972	3123///3125///3126///100507714///100507709	HLA-DRB1|HLA-DRB3|HLA-DRB4|LOC100507714|LOC100507709
17071259	1,77E-04	2,0205107	203111	C8orf47
17117542	1,77E-04	-1,3623428		

16658864	1,83E-04	1,3961703	5226	PGD
16669485	1,83E-04	-<br>1,6347804		
16727936	1,83E-04	1,4852564	4723	NDUFV1
16810514	1,83E-04	2,0584335	5479	PPIB
16829235	1,83E-04	2,0068986	353	APRT
16856757	1,83E-04	1,820403	4946	OAZ1
16863091	1,83E-04	1,6420385	5819	PVRL2
16977396	1,83E-04	2,0372362	79966	SCD5
17019338	1,83E-04	1,5157295	10591	C6orf108
17041240	1,83E-04	-3,898334	100509457///100507718///3117	LOC100509457|LOC100507718|HLA-DQA1
16685482	1,85E-04	1,8842115	79693	YRDC
16699739	1,85E-04	1,4858211	142	PARP1
16790464	1,85E-04	1,9129746	1603	DAD1
16825097	1,85E-04	1,9008319	4706	NDUFAB1
16825468	1,85E-04	1,9683814	552900///654483///440354	BOLA2|BOLA2B|LOC440354
16825683	1,85E-04	1,9670962	552900///654483///440354	BOLA2|BOLA2B|LOC440354
16839769	1,85E-04	1,5061452	100533970///30851///5026	P2RX5-TAX1BP3
16876764	1,85E-04	-<br>1,6034125	91543	RSAD2
17036504	1,85E-04	-<br>2,2638543		
16977052	1,85E-04	-6,469104	3627	CXCL10
17004974	1,86E-04	1,5184472	51406	NOL7
17083847	1,86E-04	1,6178629	10670	RRAGA
17115352	1,86E-04	1,5278205	8260///393	NAA10|ARHGAP4
16862596	1,88E-04	1,9224035	6223	RPS19
16852389	1,90E-04	-<br>2,2552292	1630	DCC
16787902	1,96E-04	8,145993	12	SERPINA3

16868130	1,96E-04	1,8387715	51293	CD320
17103784	1,96E-04	-<br>1,6398962	170685	NUDT10
16889929	2,00E-04	1,7584442		
16981266	2,00E-04	-<br>1,5013981	91351	DDX60L
16989408	2,00E-04	-<br>1,6684508	100616209	MIR4461
16681473	2,01E-04	1,922047		
16757710	2,01E-04	1,8065763	5037	PEBP1
17032870	2,01E-04	-<br>1,4926887	3135	HLA-G
17037719	2,01E-04	1,2916037	135644	TRIM40
17102632	2,01E-04	1,7150263	58526	MID1IP1
16868797	2,02E-04	1,585508	10053	AP1M2
16861047	2,08E-04	1,6497496	10430	TMEM147
16935490	2,13E-04	1,4879924	4809	NHP2L1
17103509	2,13E-04	1,8468839	10084	PQBP1
16943040	2,18E-04	-<br>1,9250103		
16673820	2,36E-04	1,6444788		
16829885	2,38E-04	2,011357	5694	PSMB6
17028338	2,41E-04	-<br>2,3941314	3119///100293977	HLA-DQB1|LOC100293977|HLA-DQB1-AS1
16856946	2,42E-04	1,955492	4782	NFIC
17090881	2,42E-04	1,5250567	266655	LINC00094
17104122	2,42E-04	2,0575356	28986	MAGEH1
17078342	2,45E-04	1,7429962	6921	TCEB1
17038622	2,49E-04	-2,413997	3119///100293977	HLA-DQB1|LOC100293977
16754324	2,51E-04	1,3948262	552889	ATXN7L3B

17040375	2,53E-04	-1,553913	3135	HLA-G
16910953	2,56E-04	1,4029346	64949	MRPS26
17113696	2,59E-04	-1,6400647	158801	NKAPP1
16875056	2,59E-04	-1,776537	55769	ZNF83
17033604	2,70E-04	-4,291426	3122	HLA-DRA
16783620	2,80E-04	-1,5098186		
16840723	2,80E-04	2,3185096	112483	SAT2
16908822	2,85E-04	1,6947323	79586	CHPF
16993268	2,85E-04	1,6858522	3182	HNRNPAB
17115669	2,88E-04	-1,7051452	30848	CTAG2
16737636	2,93E-04	2,2720232		
16863684	2,93E-04	1,9920082	6415	SEPW1
16959805	2,93E-04	1,5080291	5947	RBP1
17105524	2,93E-04	1,728715	51566	ARMCX3
16879923	3,00E-04	1,4225831	2956	MSH6
16702503	3,02E-04	1,4805772	8872	CDC123
16720392	3,02E-04	1,595438	977	CD151
17035880	3,02E-04	-4,303137	3122	HLA-DRA
16861118	3,03E-04	1,9661225	1340	COX6B1
17038596	3,03E-04	-4,3075366	3122	HLA-DRA
17041225	3,03E-04	-4,306221	3122	HLA-DRA
17007292	3,18E-04	-2,1197865	3118///100509457///100507718///3117	HLA-DQA2|LOC100509457|LOC100507718|HLA-DQA1
16808550	3,26E-04	-2,4913206	100652936	LOC100652936
16690511	3,27E-04	2,0215955	6884	TAF13

17003479	3,28E-04	1,9138018	55651	NHP2
16688339	3,31E-04	2,0328815	79971	WLS
17020731	3,38E-04	-2,5204964		
16955064	3,45E-04	2,2849724	7086	TKT
16992456	3,45E-04	1,5734212	8992	ATP6V0E1
16913263	3,45E-04	1,4530597	2036	EPB41L1
16983172	3,48E-04	1,6297066	22948	CCT5
17033646	3,48E-04	-2,2227404	3118///3117	HLA-DQA2|HLA-DQA1
16663317	3,57E-04	1,5020454	4904	YBX1
16981219	3,59E-04	-2,1647646	55601	DDX60
16776883	3,61E-04	1,6396625	55002	TMCO3
16823083	3,62E-04	1,547804	283871	PGP
16873391	3,63E-04	1,6126294	6633	SNRPD2
16775421	3,66E-04	1,6785153	7347	UCHL3
16854928	3,69E-04	1,4303629	498	ATP5A1
16822652	3,72E-04	1,2853354	115939	TSR3
16873987	3,73E-04	1,4970053	6141	RPL18
16985079	3,73E-04	1,7719582	91942	NDUFAF2
17042548	3,73E-04	-2,7842646	3113	HLA-DPA1
17081943	3,73E-04	1,501881		
16663989	3,76E-04	1,4919182	94163///26811///6202	SNORD38B|SNORD55|RPS8
16840970	3,76E-04	1,2298057	6154	RPL26
17000485	3,76E-04	1,5273952	3313	HSPA9
17115453	3,76E-04	1,3496842	4204	MECP2
16731103	3,79E-04	-1,6710529		

16775643	3,79E-04	1,4824375	54602	NDFIP2
16778559	3,79E-04	-<br>1,6915033	94240	EPSTI1
16923031	3,79E-04	-1,94473	4599	MX1
17017244	3,79E-04	1,4010814	7917	BAG6
17031139	3,79E-04	-4,12354	3122	HLA-DRA
17031887	3,79E-04	1,3989016		
17034357	3,79E-04	1,40188		
17056358	3,79E-04	1,4584978	79017	GGCT
17070242	3,79E-04	-2,767433		
17084966	3,79E-04	1,3541204	9380	GRHPR
17106604	3,79E-04	1,5992155	4694	NDUFA1
16842766	3,79E-04	1,9763907	6388	SDF2
17039400	3,79E-04	1,4002092		
17110429	3,79E-04	1,8499894	54539	NDUFB11
16858122	3,85E-04	1,555652	51073	MRPL4
16873541	3,85E-04	1,6260523	55228	PNMAL1
17017428	3,85E-04	1,2796769	7407	VARS
17028313	3,85E-04	-<br>3,9053702	3122	HLA-DRA
16788190	3,89E-04	-<br>1,4826707		
16873313	3,91E-04	1,6264542	2067	ERCC1
16868564	3,91E-04	1,7818547	8666	EIF3G
17032533	3,92E-04	-2,860152	3113	HLA-DPA1
16820076	3,92E-04	2,0519803	29100	TMEM208
17087498	3,92E-04	2,2672267	10952	SEC61B
16900148	3,94E-04	-<br>4,3953347		IGKV2-24
17018039	3,94E-04	-2,854245	3113	HLA-DPA1

17029102	3,94E-04	1,3882107		
17105010	3,96E-04	1,7143223	1349	COX7B
17073934	3,98E-04	-1,3500898		
17038629	4,10E-04	-2,084153	3118///3117	HLA-DQA2|HLA-DQA1
16714880	4,12E-04	1,8399564	56521	DNAJC12
16834540	4,15E-04	1,799594	6155	RPL27
17028326	4,17E-04	-4,290646	3117///100509457///100507718	HLA-DQA1|LOC100509457|LOC100507718
16670492	4,20E-04	1,8319719	54460	MRPS21
16693005	4,20E-04	-1,54058	6944	VPS72
16838917	4,20E-04	1,7126404	51529	ANAPC11
16858344	4,20E-04	1,4060313	6597	SMARCA4
16972824	4,20E-04	1,4285648	56977	STOX2
17100641	4,20E-04	-1,281491		
17117533	4,20E-04	-1,8627638	7704	ZBTB16
16706712	4,27E-04	1,624591	27069	GHITM
16918589	4,27E-04	1,4979943	2937	GSS
16927198	4,27E-04	1,4395759	5902	RANBP1
17005385	4,27E-04	1,6059514	55856	ACOT13
17006197	4,27E-04	1,2888417	135644	TRIM40
16791849	4,34E-04	2,0812824	171546	SPTSSA
16955975	4,54E-04	1,4500909	8801	SUCLG2
17032993	4,54E-04	-1,9589996	3133	HLA-E
17040037	4,54E-04	-2,6634483	3113	HLA-DPA1
17098071	4,54E-04	1,6792761	4702	NDUFA8
16759500	4,56E-04	1,4246106	79050	NOC4L
17105706	4,60E-04	1,7512504	51186	WBP5

16946228	4,62E-04	1,3108915	56945	MRPS22
16734570	4,63E-04	1,3121299	114879	OSBPL5
17038609	4,63E-04	-3,9529762	3117///100509457///100507718	HLA-DQA1|LOC100509457|LOC100507718
16861841	4,63E-04	1,6940037	27335	EIF3K
16669169	4,73E-04	-2,375645	914	CD2
16900098	4,75E-04	-15,859056	50802	IGK@|IGKJ3
16948909	4,75E-04	1,4795308	1981	EIF4G1
16859825	4,79E-04	1,4790732	7311	UBA52
16866951	4,79E-04	1,4345804	84823	LMNB2
16956027	4,79E-04	1,5627555	7110	TMF1
17041883	4,79E-04	1,3805054		
17054950	4,79E-04	1,2525753	27102	EIF2AK1
16666485	4,80E-04	-2,123223	10964	IFI44L
16689384	4,85E-04	-3,7952335	115361	GBP4
16733435	4,85E-04	1,4534632	334	APLP2
16775719	4,85E-04	-1,5986516		
16782082	4,85E-04	-1,8777298		TRAJ21
16872229	4,85E-04	1,5755798	6217	RPS16
16979944	4,85E-04	-2,223742		
16669632	4,86E-04	-1,4101589		
16740406	4,86E-04	-1,3577433		
16833787	4,86E-04	1,5376647	84152	PPP1R1B
16857080	4,86E-04	1,4704349	51588	PIAS4

17057990	4,86E-04	1,886516	389493	NUPR1L
17098880	4,86E-04	1,3772095	89891	WDR34
17030233	4,88E-04	1,2946566	135644	TRIM40
16864159	4,89E-04	1,3939202	6205	RPS11
17074004	4,89E-04	1,4888557		
17103572	4,89E-04	1,3165352	28952	CCDC22
16695508	4,98E-04	1,7983317	100131187///50848	TSTD1|F11R
17035037	4,98E-04	-<br>1,5422388	3135	HLA-G
16684000	5,02E-04	1,6336333	8547	FCN3
17006261	5,02E-04	-<br>1,9484694	3133	HLA-E
16821519	5,03E-04	1,5992388	9100	USP10
16717359	5,03E-04	1,6961842	2805	GOT1
16744888	5,03E-04	1,4259928	53826///100533181///486	FXYD6|FXYD6-FXYD2
16900100	5,03E-04	-4,665438		IGKV1-5
17035115	5,15E-04	-<br>1,9465622	3133	HLA-E
16972764	5,17E-04	1,7116982	3622	ING2
17103560	5,21E-04	1,7146511	5355	PLP2
16935623	5,21E-04	1,6334113	27341	RRP7A
16690638	5,24E-04	1,9852717	2947	GSTM3
17035141	5,24E-04	1,5369188	23	ABCF1
17039907	5,24E-04	-<br>2,3113365	3125///3123///3126///100507714///100507709	HLA-DRB3|HLA-DRB1|HLA-<br>DRB4|LOC100507714|LOC100507709
17040488	5,25E-04	-1,948986	3133	HLA-E
17027443	5,28E-04	-<br>1,9494969	3133	HLA-E
17027471	5,29E-04	1,510678	23	ABCF1
16751554	5,30E-04	1,6481707	3875	KRT18

16825956	5,46E-04	1,6450245	79001	VKORC1
17037774	5,47E-04	-1,9468852	3133	HLA-E
17059628	5,47E-04	1,7681941	1595///401387	CYP51A1|LRRD1
16767744	5,47E-04	-2,128821		
16771361	5,47E-04	1,8348217		
16916312	5,47E-04	1,4675679	128637	TBC1D20
16946439	5,47E-04	1,733469	9616	RNF7
17006122	5,47E-04	-1,561376	3135	HLA-G
16879276	5,58E-04	1,6322337	729967	MORN2
17040514	5,58E-04	1,5255629	23	ABCF1
16883426	5,61E-04	1,5050399	9669	EIF5B
17057884	5,61E-04	1,522783	23480	SEC61G
16943107	5,64E-04	1,6371412	131544	CRYBG3
17099498	5,68E-04	1,7097604	6834	SURF1
16733759	5,70E-04	-1,516403	283177	LOC283177
16868654	5,70E-04	1,6896214	112812///100125288	FDX1L|ZGLP1
17000460	5,70E-04	1,8826665		
17033021	5,70E-04	1,528959	23	ABCF1
16867370	5,72E-04	2,2436762	56005	C19orf10
17030288	5,72E-04	-1,9399768	3133	HLA-E
16856581	5,74E-04	1,4948384	6209	RPS15
16745026	5,78E-04	1,3933623		
16838879	5,80E-04	1,5851287	6182///1468	MRPL12|SLC25A10
16840599	5,87E-04	1,9578655	1366	CLDN7
17034714	5,89E-04	-1,5274811	3125///3127///3123///100507714///100507709	HLA-DRB3|HLA-DRB5|HLA-DRB1|LOC100507714|LOC100507709
17037800	5,89E-04	1,5354346	23	ABCF1

17063656	5,91E-04	1,4705685	51650	MRPS33
17114033	5,91E-04	-1,4397829		
17030176	5,91E-04	-1,5242691	3135	HLA-G
17091312	5,98E-04	1,3983288	85014	TMEM141
16767663	6,05E-04	1,4631851		
16941176	6,05E-04	1,3185245	29890	RBM15B
16939495	6,07E-04	1,2913595	9045	RPL14
16668109	6,09E-04	-2,6850965	677766	SCARNA2
16731945	6,12E-04	1,5293533	372	ARCN1
16738536	6,12E-04	1,7855188	26519	TIMM10
16657910	6,16E-04	1,4729313	8511///8510	MMP23A|MMP23B
16822913	6,17E-04	2,0387099	6187///26784	RPS2|SNORA64|RPS2P7|RPS2P5|RPL17P43|RPS2P55
17098411	6,29E-04	2,2478664	3309	HSPA5
16823193	6,37E-04	1,6905437	6923	TCEB2
16867511	6,37E-04	1,3496263	9361	LONP1
17019549	6,37E-04	1,7143139	64928	MRPL14
17034851	6,37E-04	-2,7524571	3113	HLA-DPA1
16821139	6,39E-04	1,3650318	22879	MON1B
17063066	6,45E-04	1,996076	1937///100500804	EEF1G|MIR3654
16701275	6,53E-04	-1,4531077		
16804942	6,58E-04	1,5855389	5045	FURIN
17006288	6,58E-04	1,4693253	23	ABCF1
16693173	6,58E-04	1,3236822	65005	MRPL9
16857897	6,63E-04	1,5994787	9230	RAB11B
17082873	6,73E-04	1,3427573	6132	RPL8

17032930	6,75E-04	1,2896377	135644	TRIM40
16845263	6,77E-04	1,7816669	8678	BECN1
17035064	6,77E-04	1,5667026	30834	ZNRD1
16751190	6,80E-04	1,7352667	25840	METTL7A
16672654	6,84E-04	-1,792749	57823	SLAMF7
16899086	6,84E-04	1,7737008	388962	BOLA3
17030316	6,84E-04	1,5254252	23	ABCF1
17070331	6,84E-04	2,0585506	92421	CHMP4C
17027187	6,87E-04	-2,7348022	3113	HLA-DPA1
16674269	6,90E-04	-1,3482178	84066	TEX35
16661533	6,92E-04	1,4870572	93974	ATPIF1
17109432	6,92E-04	1,4719306	10742	RAI2
16931400	6,97E-04	1,4238071	55687	TRMU
16700023	6,99E-04	1,4968258	64746	ACBD3
16920091	6,99E-04	-1,5425156		
16863148	7,02E-04	1,5346295	1209	CLPTM1
17073527	7,11E-04	1,5185572	54512	EXOSC4
16893992	7,13E-04	1,4896117	55256	ADI1
17037331	7,13E-04	-2,7331676	3113	HLA-DPA1
16846661	7,22E-04	1,5710038	51264	MRPL27
17032882	7,27E-04	-1,6889688	3105	HLA-A
17032389	7,31E-04	-1,5086173	3125///3127///3123///100507714///100507709	HLA-DRB3|HLA-DRB5|HLA-DRB1|LOC100507714|LOC100507709
16718493	7,35E-04	-1,3642064		

16729548	7,37E-04	1,5901824	6234	RPS28
17113448	7,37E-04	-1,6954516	3598	IL13RA2
16836131	7,42E-04	1,4675616	654364///4831///4830	NME1-NME2
16761923	7,42E-04	1,9830223		
17103804	7,42E-04	1,4660379	9500	MAGED1
16817721	7,46E-04	1,8282983	253982	ASPHD1
16684175	7,46E-04	1,799823	8420///6080	SNHG3|SNORA73A
16995957	7,50E-04	1,3802195	10605	PAIP1
16765226	7,57E-04	-1,3369949	3695	ITGB7
16804789	7,59E-04	1,6622634		
16760406	7,65E-04	1,5586406	51258	MRPL51
16797213	7,65E-04	1,3617967	55038	CDCA4
16868920	7,65E-04	1,7567555	374882	TMEM205
16940738	7,65E-04	1,5341284	327	APEH
17030207	7,65E-04	1,5364094	30834	ZNRD1
17035022	7,65E-04	-1,5157967	3134	HLA-F
17036785	7,65E-04	1,2643965		
16910645	7,69E-04	1,5378715	83541	FAM110A
16940535	7,72E-04	1,3568053	11277	TREX1
16658568	7,72E-04	1,4761195	11315	PARK7
16733858	7,72E-04	1,2966557	59307	SIGIRR
16886757	7,72E-04	1,4381158	8502	PKP4
16894361	7,72E-04	1,2200769	10971	YWHAQ
16922428	7,72E-04	1,3544208	54065	FAM165B
17026125	7,72E-04	1,5344347	30834	ZNRD1
17090155	7,74E-04	-2,246233		

16774512	7,75E-04	1,5239638	100287803	LOC100287803
17106601	7,76E-04	1,2618986		
16724235	7,76E-04	1,5763346	120071	GYLTL1B
16747612	7,76E-04	1,5496801	113246	C12orf57
16875796	7,76E-04	1,4866302	163033	ZNF579
16681477	7,82E-04	1,4103892	22883	CLSTN1
16825410	7,82E-04	1,6843439	7284///100616256	TUFM|MIR4721
16925037	7,90E-04	1,4918102	56683	C21orf59
16875848	7,97E-04	1,514407	126208	ZNF787
16871613	8,01E-04	2,4834473	5438	POLR2I
16866718	8,04E-04	1,7795888	10975	UQCR11
17049702	8,11E-04	1,476322	10467	ZNHIT1
16682042	8,16E-04	2,0886676	440567	UQCRHL
16859446	8,18E-04	1,5160171	79629	OCEL1
16895647	8,18E-04	1,7834874	10113	PREB
16840759	8,34E-04	1,6581913	84316	LSMD1
16661342	8,37E-04	1,5463104	388610	TRNP1
16873534	8,40E-04	1,4265156	83987	CCDC8
16660933	8,41E-04	1,5582517	26119	LDLRAP1
16677748	8,41E-04	1,3814014	375056	MIA3
16735734	8,41E-04	-1,5068582	100463486///100462981///100616263	MTRNR2L8|MTRNR2L2|MIR4485
16693656	8,42E-04	1,4254094	5872	RAB13
17029847	8,42E-04	-2,5091522	3113	HLA-DPA1
16661323	8,42E-04	1,6138302	10726	NUDC
16734154	8,49E-04	1,8446877	5441	POLR2L
16945320	8,49E-04	1,7005284	22820	COPG1

16675578	8,54E-04	-2,3872492	5788	PTPRC
16995601	8,58E-04	-1,9592437	2533	FYB
17022949	8,58E-04	-1,7356286		
17051659	8,58E-04	-1,5514944		
17071579	8,63E-04	-1,683079		
17118407	8,63E-04	1,3604668	100132249///643395	LOC100132249|LOC643395
16858235	8,76E-04	1,5124986	3609	ILF3
16792501	8,77E-04	1,4168247	6166	RPL36AL
16705450	8,81E-04	1,4514829	79009	DDX50
16822809	8,81E-04	1,5071676	65993	MRPS34
16860946	8,81E-04	1,6344249	51599	LSR
16878406	8,81E-04	1,5841832	9553	MRPL33
16893414	8,81E-04	1,3324766	50636	ANO7
16984801	8,81E-04	-2,8032846	3001	GZMA
17044829	8,81E-04	1,2949237	100526825///84182///11185///358	INMT-FAM188B|AQP1
16934434	8,83E-04	-1,3952016	80833	APOL3
16844207	8,83E-04	-1,5551863	22806	IKZF3
16851847	8,87E-04	-1,3405286		
16878378	8,87E-04	1,5074565	22950	SLC4A1AP
17035918	8,87E-04	-1,978456	3118///3117	HLA-DQA2|HLA-DQA1
17072222	8,87E-04	2,237585	100506980	LOC100506980
16992809	8,91E-04	1,9060568	192286	HIGD2A
17099484	8,93E-04	1,3430551	6837	MED22

16789524	8,99E-04	1,9841598		
16837754	9,20E-04	1,8571781	51081	MRPS7
17118026	9,21E-04	1,3315802		
17026808	9,24E-04	1,2493093		
16846954	9,30E-04	1,6878409	51649	MRPS23
17008534	9,30E-04	1,5907764	100188893///29964	TOMM6|PRICKLE4
17094434	9,31E-04	1,3810116	100132249///643395	LOC100132249|LOC643395
16741833	9,37E-04	1,4488616	10809	STARD10
16820572	9,42E-04	1,3903086	27183	VPS4A
16880256	9,46E-04	1,4738526	112942	CCDC104
16671381	9,49E-04	1,7039571	10456	HAX1
16896320	9,51E-04	1,456253	25940	FAM98A
16899459	9,51E-04	-1,487686		
17056968	9,51E-04	1,5037178	136647	MPLKIP
16862145	9,54E-04	1,6365764	5704	PSMC4
16948989	9,54E-04	1,4602902	5437	POLR2H
16963891	9,54E-04	1,3656112		
17032067	9,54E-04	1,2539351		
16854854	9,59E-04	-1,6034994	100422829	MIR4319
16748502	9,70E-04	1,2988685	51202	DDX47
17053775	9,70E-04	1,6863849	57180	ACTR3B
17071188	9,70E-04	1,5212528	92140	MTDH
16791167	9,74E-04	1,4505002	145553///100528064///4738	MDP1|NEDD8-MDP1
17017575	9,77E-04	1,3908607	7936///100302242	RDBP|MIR1236
16848721	9,80E-04	1,6964691	10476	ATP5H
16898578	9,88E-04	1,8060246	2673	GFPT1
16882720	9,98E-04	-9,656052		IGKV1D-39
